# Clinical effectiveness of pulsed electromagnetic field therapy as an adjunct treatment to eccentric exercise for Achilles tendinopathy: a randomised controlled trial

**DOI:** 10.1186/s13063-023-07434-6

**Published:** 2023-06-12

**Authors:** Violet Man-Chi Ko, Xin He, Sai-Chuen Fu, Patrick Shu-Hang Yung, Samuel Ka-Kin Ling

**Affiliations:** Department of Orthopaedics and Traumatology, Faculty of Medicine, The Chinese University of Hong Kong (CUHK), Shatin, Hong Kong SAR China

**Keywords:** Achilles tendinopathy, Pulsed electromagnetic field (PEMF), Eccentric exercise, Rehabilitation

## Abstract

**Background:**

The Achilles tendon is the largest and strongest tendon in the human body. Achilles tendinopathy (AT) is a common clinical problem with Achilles overuse. Eccentric exercise is often used as an initial treatment for these patients. Most patients with AT experienced moderate to severe pain, limiting the incentive to perform eccentric exercise. It is difficult for them to complete eccentric exercise for 3 months consecutively to obtain significant improvements. Using PEMF as an adjunct, there could be immediate pain relief and improved response to eccentric exercise by modulating the mechanical properties of the Achilles tendon. Participants may experience less pain while performing eccentric exercises to increase compliance with the rehabilitation programme.

**Methods:**

This prospective randomised double-blinded, placebo-controlled trial aims to investigate the treatment effects of PEMF for participants with AT. All participants are randomised into two groups: the intervention group (*n* = 20; active PEMF treatment and eccentric exercise) and the control group (*n* = 20; sham treatment and eccentric exercise). Researchers perform self-reported, functional and ultrasonographic outcomes during baseline assessment, 4 weeks, 8 weeks follow-ups, and 3 and 6 months follow-ups after the commencement of the PEMF treatment.

**Discussion:**

AT is a common clinical condition affecting athletes and sedentary populations. It is essential to investigate treatment adjuncts to improve rehabilitation outcomes for these patients. This trial may demonstrate the effectiveness of PEMF in relieving pain, improving function, and restoring mechanical changes of the tendon in participants with AT.

**Trial registration:**

ClinicalTrials.gov NCT05316961. Registered on 7th April 2022.

## Administrative information

**Table:** 

Title {1}	Clinical effectiveness of pulsed electromagnetic field therapy as an adjunct treatment to eccentric exercise for Achilles tendinopathy: a randomised controlled trial
Trial registration {2a and 2b}	ClinicalTrials.gov (NCT05316961). Registered on 7th April 2022
Protocol version {3}	Version 2
Funding {4}	This trial is fully funded by the Health and Medical Research Fund (HMRF), Food and Health Bureau of the government of HKSAR (Ref: 10210186) The study funder does not take on the legal responsibility and management of the research study. It only provides financial support to this research study
Author details {5a}	Violet Man-Chi Ko, MSc^1^, Xin He, PhD^1^, Sai-Chuen Fu, PhD^1^, Patrick Shu-Hang Yung, FRCS (Edin)^1^, Samuel Ka-Kin Ling, ChM (Edin)^1^ ^1^Department of Orthopaedics and Traumatology, Faculty of Medicine, The Chinese University of Hong Kong (CUHK), Hong Kong SAR, China
Name and contact information for the trial sponsor {5b}	The Chinese University of Hong Kong Department of Orthopaedics and Traumatology Dr Samuel Ka-kin Ling Room 74034, 5/F, Lui Che Woo Clinical Science Building, Prince of Wales Hospital, Shatin, Hong Kong SAR, China samuel.ling@link.cuhk.edu.hk
Role of sponsor {5c}	The study sponsor takes on the legal responsibility for the initiation and management of the research study, including study design, data collection and analysis, the safety of participants and publication The study funder does not take on the legal responsibility and management of the research study. It only provides financial support to this research study

## Introduction

### Background and rationale {6a}

The Achilles tendon is the largest and strongest tendon in the human body. Achilles tendinopathy (AT) is a common clinical problem with Achilles overuse; it is a clinical condition characterised by a combination of pain and swelling in the posterior leg and heel region. AT results from an altered tendon structure and mechanical properties. It impairs lower extremity function during activities of daily living and athletic capability [1]. A comprehensive treatment plan could optimise the recovery of tendon health and minimise repeated injury risk [2]. There are many limitations in the existing treatments (such as compliance problems in eccentric muscle exercises etc.), with many patients requiring major surgery, such as tendon transfers, due to poor functional improvement after conservative treatments.

The number of Achilles tendon problems has risen in industrialised countries over the past decades [1]. AT is common in athletes who participate in running and jumping sports, with a reported prevalence of 43% in elite track and field athletes and as high as 83% in middle-distance runners [3]. However, it is not purely an athletic injury, with around 65% of injuries unrelated to sports [4].

AT is treated by various interventions, including exercises, orthotics, laser therapy, manual soft-tissue mobilisation, extracorporeal shockwave therapy, injection therapies, surgical debridement, and tendon reconstruction. The treatment with the highest level of evidence is exercise rehabilitation. The exercise aims to provide a mechanical load to the tendon to promote remodelling, decrease pain, and improve calf muscle strength and lower leg function. The loading programmes have consisted of eccentric exercise, isolated concentric, or a combination of concentric and eccentric exercises. They have good reported results in controlled settings, but the effects are limited by poor compliance [2]. Most participants with AT experienced moderate to severe pain, reducing the incentive to perform eccentric exercise. It was difficult for patients to complete the training for 3 months consecutively to obtain significant improvements. Using PEMF as an adjunct, there could be immediate pain relief and improved response to eccentric exercise by modulating the mechanical properties of the Achilles tendon. Patients may experience less pain while performing eccentric exercises to increase compliance with the rehabilitation programme.

Denaro et al. investigated the effects of pulsed electromagnetic fields on human tenocyte cultures. They assessed whether pulsed electromagnetic field therapy (PEMF) could represent a viable therapeutic option in tendon pathologies. A controlled laboratory study evaluated the effects of PEMF on an in vitro tenocyte ‘‘wound’’ closure model. Primary human tenocytes were isolated from healthy supraspinatus and quadriceps tendons. An in vitro cut was mechanically produced in tenocyte culture to mimic the failed healing response in tendinopathy. Compared with the control group, a significant decrease in the ‘‘wound’’ width was found after 12 and 24 h of PEMF exposure. Exposure to PEMF significantly accelerated cut closure 12 and 24 h after the injury. The results supported that PEMF could be a healing-promoting agent in tendon injury. This kind of exogenous stimuli could accelerate the intrinsic tendon healing process by acting directly on tenocytes, the basic cellular component of the tendon tissue, and synthesise collagen and all components of the extracellular matrix. These results provide the preliminary in vitro work and the basis to support the study of the in vivo effects of PEMF on tendinopathies [5].

In addition, a parallel prospective study showed PEMF to reduce pain in patients with AT. Visual analogue scores for pain measurement decreased from 6.9 ± 1.3 at baseline to 3.6 ± 2.0 within 12 weeks [6]. However, there were some limitations in this published study. This study only measured the immediate effect of PEMF on pain in participants. There were no measurements on the functional and mechanical properties of the Achilles tendon, so no evidence supported the long-term impact in this area.

## Objectives {7}

This study aims to investigate the clinical effectiveness of PEMF as a treatment adjunct to eccentric exercise in patients with AT. The objective is to establish whether PEMF plus eccentric exercise in people with AT will improve rehabilitation outcomes compared to eccentric exercise only. The second objective is to investigate the effects of PEMF on pain, functional outcomes, and mechanical and morphological properties of tendons among patients with AT.

## Trial design {8}

This is a prospective randomised, double-blinded, placebo-controlled superiority trial with two parallel groups and a 1:1 allocation ratio to investigate the treatment effects of PEMF for participants with AT. All participants will be randomised into two groups: the intervention group (*n* = 20; active PEMF and eccentric exercise) and the control group (*n* = 20; sham PEMF and eccentric exercise). Researchers will perform all self-reported, functional, and ultrasonographic outcomes during baseline assessment, 4 weeks, 8 weeks follow-ups, and 3 and 6 months follow-ups after the commencement of the PEMF treatment.

## Methods: participants, interventions, and outcomes

### Study setting {9}

This study will be conducted at the Prince of Wales Hospital in Hong Kong which is the teaching hospital of the Chinese University of Hong Kong. The assessment and intervention will be conducted at the Prince of Wales Hospital Sports Performance and Biomechanics Laboratory.

### Eligibility criteria {10}

Participants will be recruited based on the inclusion and exclusion criteria (Table 1).

**Table 1:** Inclusion and exclusion criteria

**Inclusion criteria**
(1) Age between 18 and 70 (2) Focal clinical signs of AT with localised tenderness on palpation of the Achilles tendon (3) Recurrent complaints in 1 or both Achilles’ tendons at rest and during exercise for the preceding 3 months (4) Structural changes of the tendon were confirmed via sonographic examination during the initial physical exam (5) Informed consent
**Exclusion criteria**
(1) History of major injury or surgery on the affected lower limb in the past year (2) Mental/physical limitations hindering participant’s ability to complete assessments including severe cognitive impairment and psychiatric disorders (3) With medical or musculoskeletal problems that could affect the ability to complete assessments and intervention (i.e. pre-existing ankle arthritis, hemiplegia, etc.) (4) With active electronic implants like pacemakers and defibrillators (5) Fractures of the trained body parts within the past 12 months

All of the researchers will obtain written consent from all participants before the commencement of this study. All eligible participants will be informed about this study and given the time they need to consider participation. The investigators of this study will answer all questions from the participants. Participants who are willing to participate should sign an informed consent form. The trial will be conducted in compliance with the Declaration of Helsinki and ICH-GCP. Clinical research ethics approval is obtained from the Joint Chinese University of Hong Kong-New Territories East Cluster Clinical Research Ethics Committee (Reference number: 2021.150).

### Additional consent provisions for collection and use of participant data and biological specimens {26b}

The written consent form consists of the consent provisions for collecting and using participant data. The researcher will keep the information collected for a maximum of 5 years after publication results in PhD thesis and scientific papers. The data collected will be published to the public and in peer-reviewed scientific papers anonymously. This study will not require the use of biological specimens.

## Interventions

The intervention will be held at the Prince of Wales Hospital in Hong Kong. Participants in the intervention group will be exposed to PEMF treatment by a PEMF device (Quantum Tx, Singapore). The active PEMF does not produce heat or cause any sensation to the tissue, which allows the participants to be blinded to the treatment. Participants in the control group will receive a sham exposure with the same PEMF device. The diseased leg will be exposed to active or sham therapy for 10 min per session, and the treatment regime will run twice a week for 8 weeks, summing up 16 sessions of PEMF or sham exposure in total. The PhD candidate will be responsible to operate the PEMF machine and monitor the clinical condition of the participants. The PEMF supplier provides RFID cards generated using block randomisation to assign PEMF or sham treatment. Each participant will be allocated a unique RFID card recognisable by the PEMF machine. The PhD candidate will turn on the PEMF machine for all participants with the RFID cards. A biostatistician who does not participate in the recruitment of patients will oversee the randomisation. Hence, both participants and the research personnel are blinded, and participants will use the RFID to complete the assigned treatment without knowing which treatment they are receiving. Most participants do not report any perceivable sensations during PEMF treatment but a few participants report mild tingling or warm sensation.

The procedure of PEMF treatment is shown as follows: The subject will be seated at a 90 degrees position on a chair. The solenoids of the PEMF device will be adjusted to be over the foot and ankle (Achilles tendon and lower calf muscle). The appliance options will be adjusted to 1 mT, 10 kHz on the diseased leg for 10 min. Sham pulsed electromagnetic field therapy and eccentric exercise will be used for the treatment in the sham group. The same PEMF device and the same set of eccentric exercise will be used for the control and experimental groups. Participants will not have perceivable sensations during active or sham PEMF treatment. Therefore, assessors and participants will be blinded to the PEMF applied. 

In addition to PEMF, all participants will perform eccentric exercise. The first step is stretching exercises for the calf muscles. The stretching is a static stretch of the gastrocnemius (knee in extension) and soleus (knee in flexion). The participants are instructed to hold these for at least 30 s and repeat each exercise three times. There will be a 1-min rest between each stretch. Three sets of 10 repetitions of the eccentric exercises will be carried out once daily for 6 weeks. After 6 weeks, the participants will be instructed to carry out three sets of 10 repetitions three times per week for 6 more weeks. The intensity of the exercise should be such that pain, or discomfort, is experienced in the last set of 10 repetitions. Every session ended with the same static stretch exercise as in step 1. Suppose a participant is unable to complete three sets of 15 repetitions. In that case, the participant is instructed to start with fewer repetitions and sets (a minimum of 2 groups of 10) and progress to the total amount as able [7].

### Criteria for discontinuing or modifying allocated interventions {11b}

The treatment will be stopped, and immediate care will be provided if any significant adverse effects happen during the treatment period, including a substantial increase in the severity of pain or discomfort.

### Strategies to improve adherence to interventions {11c}

Participants may choose the time of sessions that suits them the most during working days. The opening hours of the Sports Performance and Biomechanics Laboratory will be extended to cover the evening hours so that participants may come for assessment after work. A record of compliance to eccentric exercise will be made when they come for PEMF treatment.

### Relevant concomitant care permitted or prohibited during the trial {11d}

There are no restrictions on relevant concomitant care. Participants are allowed to continue with their usual care.

### Provisions for post-trial care {30}

Participants may have access to the research clinic at the Prince of Wales Hospital if they suffer from orthopaedic conditions after the trial.

### Outcomes {12}

#### Patient-reported outcomes

The primary outcome is the score of the Victorian Institute of Sports Assessment-Achilles (VISA-A) questionnaire, which is explicitly designed for Achilles tendinopathy. VISA-A will be used to evaluate pain and symptom severity with activity in participants with AT. Chinese-speaking participants will complete the validated VISA-A HK questionnaire [8]. The VISA-A questionnaire covers three domains associated with AT: pain, function, and sporting activities. A deterioration in self-reported pain, function, and sporting activities is the critical reason patients with AT come for medical consultation. Improving questions covered in VISA-A is often the rehabilitation goal for these patients. Since PEMF was used for pain reduction for other musculoskeletal disorders, similar effects may be applied to patients with AT.

Short Form-36 questionnaires (SF-36) will be used to evaluate health-related quality of life [9]. PEMF may reduce pain levels and allow participants to resume social activities, recreational activities, and functional tasks at work. A numeric pain rating scale (NPRS) will be used to assess the level of pain on the categories “general on the day of review,” “family and responsibility at home,” “recreation,” “social activities,” and “running training or other physical activities.” Each NPRS ranges from “no pain” to “severe pain”.

#### Functional outcomes

##### Ankle range of motion

Weight-bearing lunge test will be used to measure ankle dorsiflexion’s range of motion (ROM). It will be used as an indicator of the flexibility of the gastrocnemius muscle. The participant will maximally dorsiflex the ankle while keeping the knee extended and the heel on the floor [2, 10]. The weight-bearing lunge will be performed in a standing position with the heel in contact with the ground, the knee in line with the second toe, and the great toe. Participants are allowed to place two fingers from each hand on the wall to maintain balance. They will be asked to lunge forward, directing their knees toward the wall until their knees touch the wall. The foot will progress away from the wall, and the subject will repeat the lunge until they cannot touch the wall with their knee without lifting the heel from the ground. A long ruler will be used to measure the distance between the big toe and the wall.

##### Calf muscle strength

The heel-rise test will be used to measure calf muscle strength. The participant will stand on a pressure mat (Tekscan, USA). The information on the plantar pressure will be collected for analysis [11]. Participants will be instructed to keep the knee straight and rise as high as possible on the heel each time until fatigue. They can place two fingertips per hand on the wall to maintain balance. The rhythm will be set at a frequency of 30 heel rises per minute by following a metronome [2]. This study was planned to investigate possible changes in plantar pressure distribution in participants with AT. This study hypothesises that peak pressure, midfoot maximum force, and metatarsal maximum force values in participants with AT during baseline would be lower than that after PEMF treatment. Participants could perform heel rise with better force transmission after improved pain associated with AT after PEMF treatment. Heel maximum force is hypothesised to be higher in participants during baseline assessment than after PEMF treatment. Participants with Achilles tendinopathy may have poorer eccentric control during heel raise. A sudden heel drop from the highest level of heel raise may increase the heel’s maximum force.

#### Ultrasonographic outcomes

The ultrasonographic outcomes are measured by a PhD candidate with 2 years of experience performing ultrasound measurements of the Achilles tendon. A standardised imaging procedure is used to ensure repeatability. Ultrasound examinations for this study will be performed during baseline assessment and all follow‐up visits after inclusion in the RCT. No specific instructions will be provided about activities before the consultation. The participants will be placed in a prone position on the examination table, and the ankle will be placed over the table in a relaxed position. All ultrasounds will be bilateral according to a standardised protocol using the Aixplorer machine (SuperSonic Imagine, Aix-En-Provence, France) equipped with a 12-MHz superficial linear transducer. Participants will be placed in a prone position with their legs extended. The examination will start with grayscale B mode, power Doppler ultrasound, and end with shear wave elastography.

##### Tendon thickness

All parameters are measured on the left side of the Achilles tendon and then the right side of the Achilles tendon. Greyscale ultrasound is used initially to identify the Achilles tendon. Longitudinal scans are performed medially and laterally across the Achilles tendon until the scan plane shows the Achilles tendon clearly, and maximum tendon thickness is obtained. To minimise the anisotropic effect, the transducer is adjusted so that the ultrasound beam is perpendicular to the tendon fibres. A greyscale sonographic image is taken of the Achilles tendon. Tendon thickness is measured with grayscale and axial ultrasound at the region of interest [12].

##### Neovascularity

The transducer is placed perpendicular to obtain a sagittal view of the Achilles tendon at the most painful part on palpation. The upper limit of the colour box is set on the dorsal side of the tendon. Pressure from the transducer is kept to a minimum to prevent the occlusion of neovascularization. The assessor screens the tendon for the area of maximum Doppler flow during the preparation phase for 1 min. The transducer is gently moved to medial and lateral over where Doppler flow is present. When the location of the maximum Doppler flow is identified, a sonographic image is taken. The modified Öhberg score from 0 to 4 + is determined during the US examination. In this scoring system, scores are determined as 0 (no vessels), 1 + (1 vessel, primarily anterior to the tendon), 2 + (1 or 2 vessels throughout the tendon), 3 + (3 vessels throughout the tendon), or 4 + (more than three vessels throughout the tendon). A higher score indicates more Doppler flow in the peritendinous and intratendinous tissues [13].

##### Tendon elasticity

Shear wave elastography (SWE) quantifies soft tissue stiffness and provides an absolute value of tendon stiffness [12]. Once the optimal scan plane of the tendon is identified, the SWE function is activated. SWE is set at the penetration mode with the measurable range of stiffness standardised at 0–600 kPa. The SWE colour map (height × width: 1.4 cm × 1.3 cm) is placed just above the superior border of the calcaneum. Electrogram is obtained and stored when the colour signals become steady for five seconds. Tendon stiffness is measured with the stiffness measurement tool—Q-box. The circular measurement area (Q-box) is set to 2 mm in diameter, dependent on the tendon size, and covers the Achilles tendon without including other adjacent soft tissues. Q-box is placed at the middle part of the tendon within the SWE colour map. For each Q-box, the mean (in kPa) of the stiffness of the tendon is measured [14].

The ultrasound normalisation procedure for subjects with bilateral tendinopathy will be done by using the mean values measured in healthy individuals as stated in current evidence. The mean value of a healthy Achilles tendon was reported to be 5.3 mm [15]. No vessels appear throughout the Achilles tendon for a healthy tendon (Öhberg score 0) [16]. The range of tendon elasticity measured in shear wave elastography in men and women are 657.1–766.4667 kPa and 433.5677–782.8667 kPa, respectively [17].

### Participant timeline {13}

Table 2 is used to demonstrate the schedule of enrolment, interventions, assessments, and visits for participants.

**Table 2:** Participant timeline

	Study period						
	Enrolment	Allocation	Post-allocation				
Timepoint	Before allocation	0	Week 1	Week 4	Week 8	3 months	6 months
Enrolment:							
Eligibility screen	X						
Informed consent	X						
Allocation		X					
Interventions:							
Control group: Sham PEMF plus eccentric exercise			X	X	X		
Experimental group: Active PEMF plus eccentric exercise			X	X	X		
Assessments:							
Self-reported questionnaires (NPRS, VISA-A, SF-36)			X	X	X	X	X
Functional outcomes (AROM, heel-rise)			X	X	X	X	X
Ultrasonographic outcomes (Tendon thickness, neovascularity, tendon elasticity)			X	X	X	X	X

*AROM* Ankle range of motion, *NPRS* Numeric pain rating scale, *SF-36* Short-form 36, *VISA-A* Victorian Institute of Sports Assessment-Achilles

### Sample size {14}

A minimum clinically significant difference is 16 points on the VISA-A scores based on the pilot study using VISA-A as the primary outcome in treating AT [18]. It was reported in a feasibility study using another conservative treatment, laser therapy as an adjunct treatment to eccentric exercise therapy for AT. A total of 20 participants were recruited and VISA-A was the primary outcome. Significant improvements were shown in both treatment groups in VISA-A [18]. The power calculation to detect the minimum clinically significant difference is conducted using the G Power programme at a type I error of 5%, power of 80% (correlation value, 0.03; pooled SD, 20.25). Twenty subjects per group are obtained to detect a significant difference in VISA-A among two groups if a two-way repeated-measures analysis of variance (ANOVA) is proposed. It is expected to have a 20% dropout rate. Thus, the final study sample size is 40 subjects in total [19].

### Recruitment {15}

Participants will be recruited from the Department of Orthopaedics and Traumatology at the Prince of Wales Hospital in Hong Kong. The orthopaedic surgeons will explain the details of the trial based on the eligibility criteria. They will screen all patients with Achilles tendinopathy coming for consultation in the outpatient department at the Prince of Wales Hospital. In addition, the information of the study will be sent to the coaches of the sports teams at CUHK. Recruitment information for the trial will also be sent through the internal mass mail system at CUHK. Individuals will come for eligibility assessment at the Sports Injury and Biomechanics Laboratory if they are interested to join the trial.

## Assignment of interventions: allocation

### Sequence generation {16a}

The randomisation is performed using an online research randomiser (https://www.randomizer.org/). Participants will be randomised into 1:1 allocation in a block of 10. Each allocation will be assigned a unique RFID generated by the chief engineer from Quantum TX and they will be recognisable by the PEMF machine.

### Concealment mechanism {16b}

The randomisation is performed using an online research randomiser by the chief engineer at Quantum TX. He will only share the randomisation results with the biostatistician at the Chinese University of Hong Kong. It will not be disclosed to the participants and assessors of this trial before the end of the treatment period. The participants will be assigned a unique RFID by which the PEMF or sham treatment will be randomly assigned to the RFID. Participants and assessors are blinded as participants will use the RFID to complete the treatment without knowing the treatment they receive.

### Implementation {16c}

The chief engineer from Quantum TX performs the randomisation. The randomisation result is shared with the biostatistician at the Chinese University of Hong Kong. The orthopaedic surgeon and PhD candidate from CUHK recruit participants. The PhD candidate will complete the assessment and assigns participants to interventions. The randomisation results will only be disclosed to the orthopaedic surgeon and PhD candidate after the participants complete all PEMF sessions.

## Assignment of interventions: blinding

### Who will be blinded? {17a}

Assessors and participants will be blinded to the interventions. Participants will be blinded to the PEMF given to them as active and sham PEMF will not produce heat or other perceivable sensations. The engineer performs the randomisation from Quantum TX, and it will not be disclosed to the assessors before the end of the PEMF treatment period.

### Procedure for unblinding if needed {17b}

There will be unblinding of the participants and assessors if a severe adverse event occurs. The adverse event will be reported to the Joint Clinical Research Ethics Committee of the Chinese University of Hong Kong and the New Territories East Cluster of the Hospital Authority.

## Data collection and management

### Plans for assessment and collection of outcomes {18a}

The collection of demographic data will be done during screening and before recruitment. Participants that fulfil all the inclusion criteria will be randomised into PEMF or sham groups. Follow-up assessments with various outcome parameters will be conducted with data processing details as shown in the section “Outcomes {12}”.

### Plans to promote participant retention and complete follow-up {18b}

Participants will choose the time for each PEMF session during the week. The working hours will be extended to the evenings so that participants may come for assessment and treatment after work.

### Data management {19}

All data collected will be entered into the online database in Excel files and SPSS files. All data will be kept in password-protected computers and destroyed 5 years after publication in PhD thesis and peer-reviewed journals. The PhD candidate will be responsible for data collection, data entry, and data analysis. She will enter the data into the database for screening and randomisation purposes. After data collection, she will enter all data into Excel files and then input them into SPSS files for data analysis. Double data entry and range checks for data values will be used to promote data quality.

### Confidentiality {27}

All personal information and consent forms will be stored in locked cabinets at the Sports Performance and Biomechanics Laboratory. The online electronic database with personal information will be kept on password-protected computers. All data collected will be kept strictly confidential and only accessed by members of the trial team. Participants will not have the right to access the data set. There is no plan to share the data collected with other organisations.

### Plans for collection, laboratory evaluation, and storage of biological specimens for genetic or molecular analysis in this trial/future use {33}

Not applicable. This study will not require the use of biological specimens.

## Statistical methods

### Statistical methods for primary and secondary outcomes {20a}

Statistical analysis will be performed using SPSS software (SPSS 26.0) based on the intention-to-treat principle. The normality of the data will be tested using the Kolmogorov–Smirnov test. A repeated-measures two-way analysis of variance (ANOVA) will be used to compare muscle strength, ankle range of motion, and results of questionnaires at baseline, 4 weeks, 8 weeks, and 3-, and 6-month follow-up. A nonparametric Mann–Whitney *U* test will be used to compare questionnaire results (ordinal data) between control and experimental groups. Subgroup analysis for participants with mid-portion or insertional AT will also be performed using interaction tests from ANOVA. Significant differences for each test measuring physical function will be based on an alpha level set at 0.05.

### Interim analyses {21b}

An interim analysis will be performed on the primary endpoint when 20% of participants have completed the eight weeks of PEMF. The interim analysis ensures that there is no serious adverse event that happened during the study period.

### Methods for additional analyses (e.g. subgroup analyses) {20b}

The heterogeneity in tendinopathy will be addressed by sub-group analysis. AT will be categorised into midportion and insertional tendinopathy. The assessor will make the diagnosis of the type of Achilles tendinopathy. Midportion AT will be diagnosed by local thickening and a colour doppler flow of at least two out of four in the modified Öhberg score 2–6 cm proximal to the insertion of the Achilles tendon. Insertional AT will be diagnosed by local thickening and colour Doppler flow of at least grade two of four in the modified Öhberg score within 2 cm of the insertion of the Achilles tendon. Sub-group analysis will be performed to understand if PEMF has clinical effects on all outcome measures in the midportion tendinopathy and insertional tendinopathy, respectively, since the number of participants will be smaller in the subgroups. A non-parametric test, the Mann–Whitney *U* test, will be used to detect if there are significant differences in the outcomes for each subgroup.

### Methods in analysis to handle protocol non-adherence and any statistical methods to handle missing data {20c}

The intention-to-treat analysis will be used to include all study participants in the groups to which they are randomised, regardless of any departures from the original assigned group. The missing values will be estimated by multiple imputations with the assumption that values are missing at random.

### Plans to give access to the full protocol, participant-level data, and statistical code {31c}

The protocol has been shown on ClinicalTrials.gov (ID: NCT05316961). The participant-level data and statistical code can be provided upon request.

## Oversight and monitoring

### Composition of the coordinating centre and trial steering committee {5d}

The principal investigator will monitor the progress of the study. The trial steering committee are research team members who will participate in participant recruitment, data collection, and management.

### Composition of the data monitoring committee, its role, and reporting structure {21a}

The trial steering committee consists of orthopaedic surgeons, a physiotherapist, and a biostatistician. The orthopaedic surgeons and physiotherapists will participate in data collection and analysis. The biostatistician will monitor data collection and perform interim analysis. There is no independent data monitoring committee as this is not required by the ethics committee. This is a low-risk study as PEMF was implemented as a conventional conservative treatment for patients with other musculoskeletal disorders.

### Adverse events reporting and harms {22}

The PhD candidate will have thorough training on the operation of the machine, familiarise herself with the troubleshooting scheme, and closely monitor adverse effects during PEMF. In the event of a severe adverse reaction to the PEMF, it will be reported to the trial steering committee immediately. The participant will be removed from the trial and referred to the outpatient clinic at the Prince of Wales Hospital.

An independent auditor from the ethics committee will conduct the trial conduct annually. The trial steering group meet weekly to report research progress to the principal investigator. The principal investigator will complete a standardised progress report form for the ethics committee to review conduct annually during the trial period.

### Plans for communicating necessary protocol amendments to relevant parties (e.g. trial participants, ethical committees) {25}

The investigators will apply for protocol amendments to the ethics committee and should be approved by the ethics committee before implementation. Trial participants will be informed about the amendments to the protocol. The protocol changes will be published in the Trial Register.

## Dissemination plans {31a}

The early findings of the research will be presented at the Hong Kong Orthopaedic Association annual congress, the flagship event attended by all local orthopaedic surgeons. Final results will be presented at regional sports medicine conferences such as the Asia–Pacific Knee Arthroscopy and Sports Medicine (APKASS) Congress and published in a scientific peer-review journal. The funder does not pose any restrictions on the decision of publication.

## Discussion

AT is a clinical syndrome characterised by pain, swelling, and functional impairment, indicating a degenerative inflammatory process in the Achilles tendon. The overall incidence rate of AT was 2.45 per 1000 in the adult population between 21 and 60 years in their lifetime. It can affect athletes and non-athletes. Patients with AT commonly refer to persistent pain that may result in loss of function and activity cessation. The pathogenesis of tendinopathy can be described as failed healing of the diseased tendon. Failed recovery mainly refers to prolonged inflammation and failed resolution of the normal healing process. Furthermore, increased focal vascularity contributes to the clinical presentations of chronic tendon pain, stiffness, and weakness.

An increase in tendon thickness is often found in patients with AT. It could happen in either midportion or insertion of the Achilles tendon, which is caused by swelling of the affected area. Moreover, the ingrowth of new blood vessels into tendinopathic regions is often seen in ultrasound imaging. PEMF may reduce tendon inflammation, thus reducing tendon thickness and neovascularity. Tendon elasticity is measured by shear wave elastography (SWE). SWE could quantify soft tissue stiffness and provide an absolute value of the stiffness of the soft tissue. There may be an increase in tendon stiffness in patients with AT. This is because patients may reduce the level of activities because of pain associated with AT. PEMF may minimise tendon pain and facilitate the performance of the eccentric exercise. Thus, there may be a reduction in tendon elasticity.

This trial may demonstrate the effectiveness of PEMF in relieving pain, improving function, and restoring mechanical changes of the tendon in patients with AT. AT is a common clinical condition affecting both athletes and sedentary populations. Exercise prescription is used as a non-invasive therapy for patients with AT. However, some participants do not respond well to exercise therapy and conventional treatments. It is essential to investigate treatment adjuncts to improve rehabilitation outcomes for this group of patients.

## Trial status

Enrolment in the study started on 1 July 2021. Recruitment is expected to be completed by the end of September 2023.

## Data Availability

All investigators will have access to the final trial dataset upon request. Any data required to support the protocol can be supplied on request.

## Abbreviations

ANOVA: Analysis of variance
AROM: Ankle range of motion
AT: Achilles tendinopathy
CUHK: The Chinese University of Hong Kong
NPRS: Numeric pain rating scale
PEMF: Pulsed electromagnetic field therapy
RCT: Randomised controlled trial
SF-36: Short-form 36
SWE: Shear wave elastography
VISA-A: Victorian Institute of Sports Assessment-Achilles

## References

[1] Järvinen TA, Kannus P, Maffulli N, Khan KM (2005). Achilles tendon disorders: aetiology and epidemiology. Foot Ankle Clin.

[2] Silbernagel KG, Hanlon S, Sprague A (2020). Current clinical concepts: conservative management of Achilles tendinopathy. J Athl Train.

[3] Janssen I, van der Worp H, Hensing S, Zwerver J (2018). Investigating Achilles and patellar tendinopathy prevalence in elite athletics. Res Sports Med.

[4] de Jonge S, de Vos RJ, Van Schie HT, Verhaar JA, Weir A, Tol JL (2010). One-year follow-up of a randomised controlled trial on added splinting to eccentric exercises in chronic midportion Achilles tendinopathy. Br J Sports Med.

[5] Denaro V, Ruzzini L, Barnaba SA, Longo UG, Campi S, Maffulli N (2011). Effect of pulsed electromagnetic fields on human tenocyte cultures from supraspinatus and quadriceps tendons. Am J Phys Med Rehabil.

[6] Gerdesmeyer L, Saxena A, Klueter T, Harrasser N, Fullem B, Krath A (2017). Electromagnetic transduction therapy for Achilles tendinopathy: a preliminary report on a new technology. J Foot Ankle Surg.

[7] Stasinopoulos D, Manias P (2012). Comparing two eccentric exercise programmes for the management of Achilles tendinopathy. A pilot trial. J Bodyw Mov Ther.

[8] Ko MC, Lau NN, Qiu JH, Fu SC, Yung PSH, Ling SKK (2022). Cross-cultural adaptation of Chinese Victorian Institute of Sports Assessment – Achilles (VISA-A) questionnaire for Achilles tendinopathy. Foot Ankle Orthop.

[9] Li L, Wang HM, Shen Y (2003). Chinese SF-36 Health Survey: translation, cultural adaptation, validation, and normalisation. J Epidemiol Community Health.

[10] Alshami AM, Alhassany HA (2020). Girth, strength, and flexibility of the calf muscle in patients with knee osteoarthritis: a case–control study. J Taibah Univ Med Sci.

[11] Zammit GV, Menz HB, Munteanu SE (2010). Reliability of the TekScan MatScan(R) system for the measurement of plantar forces and pressures during barefoot level walking in healthy adults. J Foot Ankle Res.

[12] Ying M, Yeung E, Li B, Li W, Lui M, Tsoi C-W (2003). Sonographic evaluation of the size of Achilles tendon: the effect of exercise and dominance of the ankle. Ultrasound Med Biol.

[13] Vlist A, Winters M, Weir A, Ardern CL, Welton N, Caldwell D (2020). Which treatment is most effective for patients with Achilles tendinopathy? A living systematic review with network meta-analysis of 29 randomised controlled trials. Br J Sports Med.

[14] Petitpierre F, Perez J-T, Bise S, Fournier C, Hauger O, Dallaudiere B (2018). Quantitative elastography of Achilles tendon using Shear Wave Elastography (SWE): correlation with zonal anatomy. Muscles Ligaments Tendons J (MLTJ).

[15] Ohberg L, Lorentzon R, Alfredson H (2001). Neovascularisation in Achilles tendons with painful tendinosis but not in normal tendons: an ultrasonographic investigation. Knee Surg Sports Traumatol Arthrosc.

[16] Watson J, Barker-Davies RM, Bennett AN, Fong DTP, Wheeler PC, Lewis M (2018). Sport and exercise medicine consultants are reliable in assessing tendon neovascularity using ultrasound Doppler. BMJ Open Sport Exerc Med.

[17] Chen B, Cheng X, Dorthe EW, Zhao Y, D'Lima D, Bydder GM (2019). Evaluation of normal cadaveric Achilles tendon and enthesis with ultrashort echo time (UTE) magnetic resonance imaging and indentation testing. NMR Biomed.

[18] Tumilty S, Munn J, Abbott JH, McDonough S, Hurley DA, Baxter GD (2008). Laser therapy in the treatment of Achilles tendinopathy: a pilot Study. Photomed Laser Surg.

[19] Tumilty SP, McDonough SP, Hurley DAP, Baxter GDD (2012). Clinical effectiveness of low-level laser therapy as an adjunct to eccentric exercise for the treatment of Achilles’ tendinopathy: a randomized controlled trial. Arch Phys Med Rehabil.

